# Transcriptional profiling of GBM invasion genes identifies effective inhibitors of the LIM kinase-Cofilin pathway

**DOI:** 10.18632/oncotarget.2412

**Published:** 2014-09-05

**Authors:** Jun-Bum Park, Sameer Agnihotri, Brian Golbourn, Kelsey C. Bertrand, Amanda Luck, Nesrin Sabha, Christian A. Smith, Sara Byron, Gelareh Zadeh, Sidney Croul, Michael Berens, James T. Rutka

**Affiliations:** ^1^ Arthur and Sonia Labatt Brain Tumor Research Centre, Hospital for Sick Children, Toronto, ON. Canada; ^2^ Department of Neurological Surgery, Ulsan University Hospital, University of Ulsan College of Medicine, Ulsan, Republic of Korea; ^3^ Division of Neurosurgery, Toronto Western Hospital, University of Toronto, Canada; ^4^ Cancer and Cell Biology Division, Translational Genomics Research Institute, Phoenix, Arizona, United States of America; ^5^ Department of Surgery, University of Toronto, Toronto ON. Canada

**Keywords:** glioblastoma multiforme, invasion, migration, Rho-GTPase, LIM kinase

## Abstract

Malignant gliomas are highly proliferative and invasive neoplasms where total surgical resection is often impossible and effective local radiation therapy difficult. Consequently, there is a need to develop a greater understanding of the molecular events driving invasion and to identify novel treatment targets. Using microarray analysis comparing normal brain samples and mesenchymal glioblastoma multiforme (GBM), we identified over 140 significant genes involved in cell migration and invasion. The cofilin (CFL) pathway, which disassembles actin filaments, was highly up-regulated compared to normal brain. Up-regulation of LIM domain kinase 1 and 2 (LIMK1/2), that phosphorylates and inactivates cofilin, was confirmed in an additional independent data set comparing normal brain to GBM. We identified and utilized two small molecule inhibitors BMS-5 and Cucurbitacin I directed against the cofilin regulating kinases, LIMK1 and LIMK2, to target this pathway. Significant decreases in cell viability were observed in glioma cells treated with BMS-5 and Cucurbitacin I, while no cytotoxic effects were seen in normal astrocytes that lack LIMK. BMS-5 and Cucurbitacin I promoted increased adhesion in GBM cells, and decreased migration and invasion. Collectively, these data suggest that use of LIMK inhibitors may provide a novel way to target the invasive machinery in GBM.

## INTRODUCTION

Cancer invasion remains a significant cause of patient related morbidity and mortality, and poses challenges for locally directed therapies such as surgery and radiation therapy. The molecular mechanisms underlying cancer invasion are not completely understood, but include the elaboration of proteolytic enzymes by tumour cells which degrade extracellular matrix (ECM) macromolecules, tumour cell:tumour cell and tumour cell:ECM interactions, and dysregulation of intrinsic molecular motors of cancer cells which result in rearrangements of the actin cytoskeleton and subsequent enhanced cancer cell motility. Recently, we have focused our research efforts on the Rho GTPase family of molecular motors in the study of glioblastoma multiforme (GBM), a highly infiltrative and invasive cancer of the brain [1–3]. We have shown that GBM cells demonstrate both mesenchymal migration predominantly through Rac1 activation and amoeboid migration through Rho/Rho kinase (ROCK) activation [4, 5].

Downsteam members of the Rho-GTPase pathway are critical intracellular mediators of the actin-modeling events that control directional cell migration and are frequently dysregulated in numerous neoplasms including GBM [3, 6–9]. Previously, we and others have demonstrated that dyregulated phosphorylation of the downstream protein Cofilin (CFL) significantly increased tumour migration and invasion *in vivo* [10–13].

CFL phosphorylation is dynamically regulated by LIM kinases (LIMK) and testis-specific kinases (TESK1 and TESK2) that phosphorylate CFL at serine-3 (S3) residues that inactivate CFL by blocking CFL's actin binding ability [14–16]. The phosphatases Slingshot and Chronophilin activate CFL through localization dependent dephosphorylation [17]. The factors known to phosphorylate and dephosphorylate CFL to enable CFL to work on downstream effector molecules leading to cell migration collectively comprise the CFL pathway.

Given that LIMK1 is a downstream effector of both the Rac and Rho pathways, which respectively regulate mesenchymal and amoeboid migration, LIMK is likely a key regulator in both modes of cell migration. Interestingly, abnormal expression of LIMK has been implicated in numerous malignancies such as prostate cancer, invasive breast cancer and melanoma [18–21].

In the current study, we identified aberrant LIMK in a gene expression array of invasion/migration genes comparing normal brain to samples from highly malignant and invasive GBM. Here we investigate the role of LIMK in GBM migration and invasion and evaluate if LIMK small molecule inhibitors are viable candidates for preclinical targeting of GBM invasiveness. To our knowledge, an in-depth study of the role of LIMK in glioma motility and invasion has not been performed previously.

## RESULTS

### Identification of Cofilin pathway dysregulation in GBM

**Figure 1:**
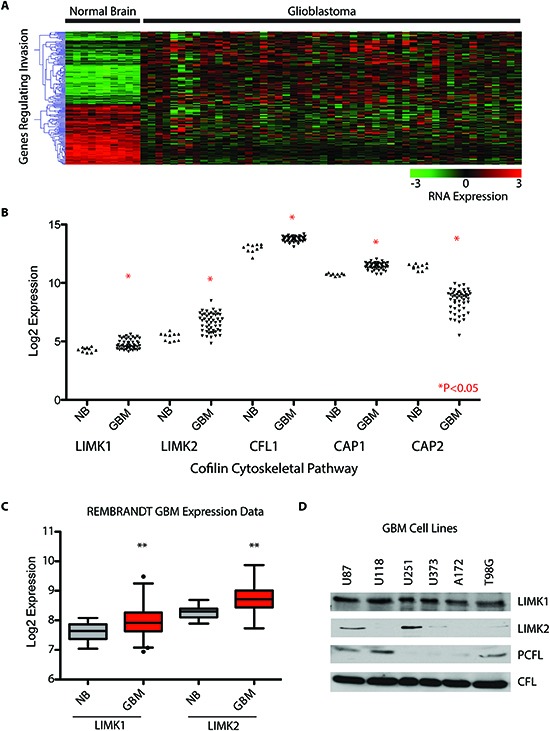
Identification of Cofilin pathway dysregulation in GBM **(A)** 700 Probe sets were investigated representing 400 genes involved in migration and invasion. Using Sam-Pairwise analysis, a fold change of 1.5 was used, p<0.05 and a Q value of <0.01. 141 Genes were identified as significantly up or down regulated compared in mesenchymal glioblastoma (n=51) versus normal brain (n=10) **(B)** Invasion Pathway Analysis identified significant deregulation of the Cofilin Pathway **(C)** LIMK1 and LIMK2 which phosphorylate CFL are up-regulated in GBM using the REMBRANDT brain tumor data set. **(D)** CFL is upregulated in GBM and LIMK1 and 2 are present in GBM cells. *p<0.05, **p<0.01.

Using gene-expression data from The Cancer Genome Atlas data set (TCGA) on the Affymetrix U133 platform we performed microarray analysis comparing 10 normal brain samples versus 51 mesenchymal GBMs. We initially selected one subtype of GBM, the mesenchymal GBM, in our discovery screen to reduce the impact of GBM subtype heterogeneity. The mesenchymal subtype also lacks immediate actionable targets, and is associated with a poor prognosis [22–24]. We compared 400 invasion/migration genes – using the gene-ontology terms invasion and migration – represented by 700 probe-sets. We identified over 141 significant genes with a 1.5 fold change (p-value < 0.05, and a false discovery rate q < 0.05) compared to normal brain (**Figure 1A**). Of the 141 genes, the cofilin pathway, which disassembles actin filaments (namely LIMK1, LIMK2, CFL, CAP1) was highly upregulated compared to normal brain (**Figure 1B, P<0.05**). Of great interest we identified up-regulation of LIM domain kinase 1 and 2 (LIMK1/2) that phosphorylates and inactivates CFL in an additional data set comparing normal brain to GBM (**Figure 1C).** Lastly, we observed robust expression of LIMK1 in several well-characterized GBM cell lines (U87, T98G and U118) and phospho-CFL in cell lines that expressed LIMK1 (**Figure 1D**). All phospho-CFL lines expressed LIMK1, but we did not observe phospho-CFL positive cell lines that were LIMK1 negative (Figure 1D).

### LIMK is of prognostic value

**Figure 2:**
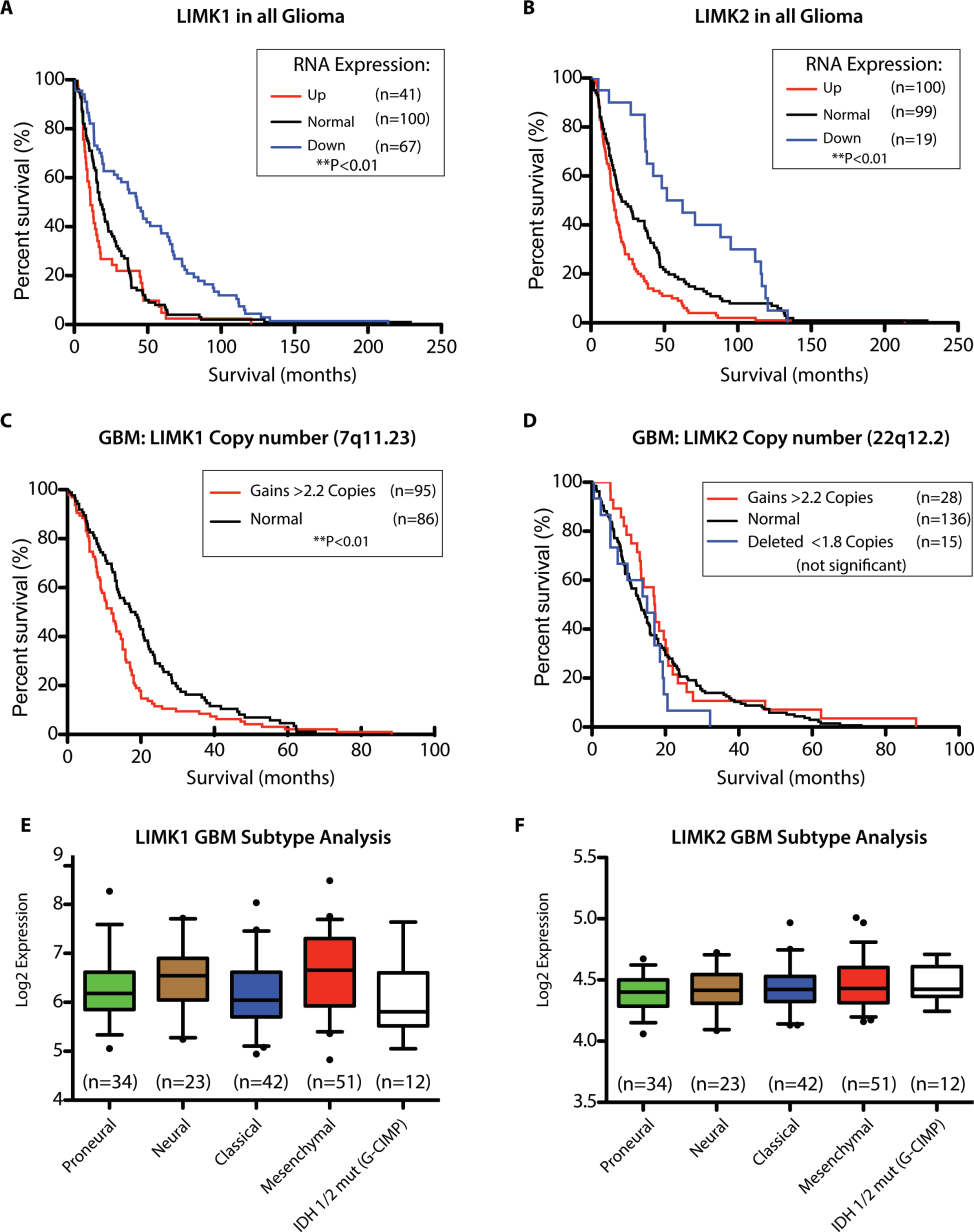
LIMK is of prognostic value **(A)** Patients with low LIMK1 in all glioma have a better overall survival at the RNA level (<2 fold compared to normal brain) in the REMBRANDT glioma data set. **(B)** Patients with low LIMK2 in all glioma have a better overall survival at the RNA level (<2 fold compared to normal brain) in the REMBRANDT glioma data set. **(C&D)** At the Copy Number Level, Patients with high LIMK1 but not LIMK2 (>3 copies compared to normal brain) have poor survival in GBM patients. **(E&F)** Using the TCGA data set for 162 samples with subtype information, LIMK1 and LIMK2 are not differentially expressed in GBM subtypes. *p<0.05, **p<0.01.

CFL and its role in migration and invasion have been previously implicated in GBM biology, however the role and potential prognostic value of its upstream regulators, LIMK1/2, are still incompletely elucidated. Towards this end, we queried if LIMK1 and LIMK2 had prognostic value in a clinically annotated dataset (REpository of Molecular BRAin Neoplasia DaTa/REMBRANDT) [25]. Based on gene expression, glioma patients (grades II–IV) with downregulated LIMK1 and LIMK2 had significantly better overall survival (**Figures 2A-B, p>0.05**). We next queried the prognostic value of LIMK1 and LIMK2 in GBM – the worst outcome group. Using DNA copy number analysis, patients with LIMK1 gains (> 3 copies of a gene) but not LIMK2 had a worse overall survival (**Figures 2C-D**, p < 0.05). Lastly, as GBM is comprised of 5 molecular subtypes (Proneural, Neural, Classical, Mesenchymal, and the G-CIMP positive subgroup), we compared LIMK1 and LIMK2 among the varying subgroups of GBM from the TCGA dataset and observed no subtype differences (**Figures 2E-F**). In summary, LIMK1 and LIMK2 down-regulation correlates with better overall survival in glioma patients; and DNA copy number gains of LIMK1 correlate with worse survival in GBM patients.

### LIMK1 and pCFL are expressed in the periphery of GBM

**Figure 3:**
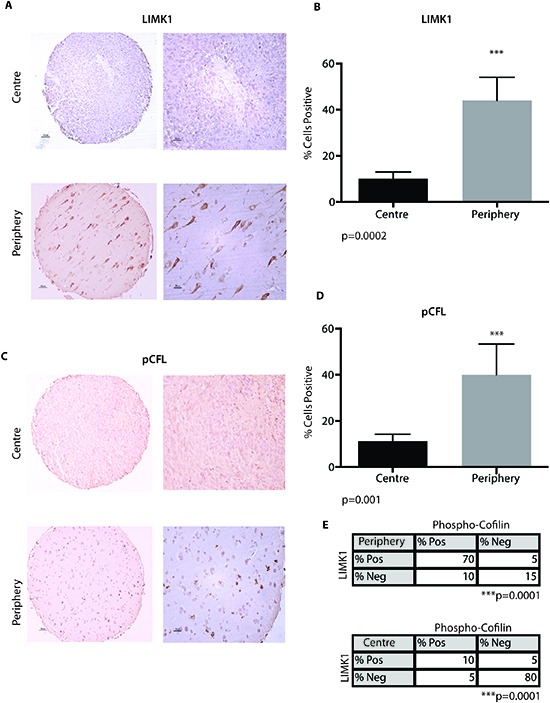
LIMK1 and pCFL are expressed in the periphery of GBM **(A)** A tissue microarray consisting (TMA) of patient matched centre and periphery samples (n=20) was stained for LIMK1 by immunohistochemical analysis (IHC). Samples were scored as positive or negative. **(B)** Ten field of views per sample were taken to quantify % percent of cells positive for LIMK1 in the centre portion of GBM or periphery of the tumour. **(C)** A TMA consisting of patient matched centre and periphery samples (n=16) was stained for pCFL by IHC. Samples were scored as positive or negative. **(D)** Ten field of views per sample were taken to quantify % percent of cells positive for pCFL in the centre portion of GBM or periphery of the tumour. ***p<0.001. **(E)** Co-expression analysis of pCFL and LIMK1 from serial IHC section of matching cores (n=5) in the centre and periphery of the tumour. pCFL and LIMK1 are positively co-expressed in tumor periphery cells (p=0.0001). pCFL and LIMK1 are negative in the same centre GBM tumor cells (p=0.0001).

GBMs are highly heterogeneous cancers phenotypically and genotypically characterized by cells demonstrating central pseudopalisading and infiltrative tumor cells at the interface between solid tumour and normal brain. Spatial and regional heterogeneity of many markers of GBM including oncogenes have been reported; however the spatial heterogeneity of LIMK1 and its target CFL are poorly characterized. Using a well-characterized tissue microarray of 20 GBM patients for which a tumour core and a matched tumour periphery sample were available we undertook immunohistochemical (IHC) analysis of LIMK1 and pCFL. We observed significant expression of LIMK1 at the periphery of the GBMs compared to the core (11/20 in the periphery versus 2/20 in the core) (**Figure 3A**). On average 43% of the cells stained positively for LIMK1 in the periphery compared to only 12% of cells that were positive in the tumour core (**Figure 3B**, p<0.05). Similarly, we observed significantly more expression of p-CFL in the periphery of the GBM compared to the core (7/16 in the periphery versus 2/16 in the core) (**Figure 3C**). On average 42% of the cells stained positively for p-CFL in the periphery compared to only 15% of cells that were positive in the tumour core (**Figure 3D**, p<0.05). We next performed co-expression analysis in which we had matching LIMK1 and pCFL protein expression from the same cells in the centre and periphery (from serial sections of our IHC analysis of LIMK1 and pCFL). We observed a correlation between positive pCFL and positive LIMK1 expression in the tumour periphery, (**Figure 3E**, Fischer's Exact Test, p=0.0001). Likewise, we observed a correlation between negative pCFL and negative LIMK1 expression in the tumor centre, (**Figure 3E**, p=0.0001).

### Phospho-cofilin is attenuated by a LIM kinase inhibitor and Cucurbitacin

**Figure 4:**
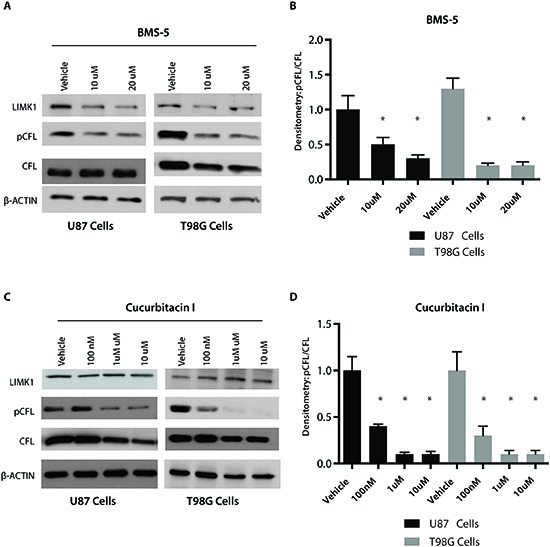
Phospho cofilin is attenuated by a LIM kinase inhibitor and Cucurbitacin I **(A)** Western blot analysis to assess the effect of LIMK1/2 inhibitor (BMS-5) on phospho cofilin (pCFL) and total cofilin at 10 uM and 20 uM. **(B)** Densitometric analysis using chemiluminescence to identify effect of BMS-5 on pCFL levels. pCFL was normalized to total CFL and a saturated blot is shown in A. **(C)** Western blot analysis to assess the effect of pCFL inhibitor (Cucurbitacin I) on phospho cofilin (pCFL) and total cofilin at 100 nM and 1 uM. **(D)** Densitometric analysis using chemiluminescence to identify effect of Cucurbitacin I on pCFL levels. pCFL was normalized to total CFL and a saturated blot is shown in C, *p<0.05.

Currently there is a lack of therapeutic agents targeting invasion and migration in GBM and there is a need for actionable targets involving these pathways. Having established that pCFL plays an integral role in GBM invasion, we explored how to target this pathway using small molecule inhibitors [12, 13, 26]. We tested LIMK1/2 and p-CFL inhibitors, BMS-5 and Cucurbitacin I. Both have been shown to inhibit phosphorylation of CFL with BMS-5 inhibiting LIMK1/LIMK2 and Cucurbitacin I inhibiting cofilin phosphorylation through an unknown mechanism. We tested both compounds on two well-established GBM cell line models, U87 and T98G cell, both of which express LIMK1 and p-CFL. Both BMS-5 and Cucurbitacin I significantly inhibited cofilin phosphorylation at 10μM and 100nM respectively in U87 cells, using linear protein quantification by chemiluminescent densitometry (**Figure 4A-B, p<0.05**). Similar results for both BMS-5 and Cucurbitacin I were seen in T98G cells where cofilin phosphorylation was inhibited at 10μM and 100nM respectively and was significant based on linear protein quantification by chemiluminescent densitometry (**Figure 4C-D, p<0.05**).

### A LIM kinase inhibitor and Cucurbitacin I inhibit viability of GBM cells

**Figure 5:**
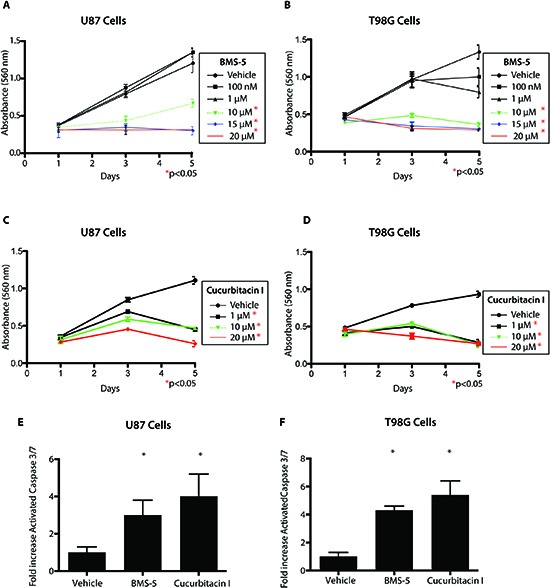
A LIM kinase inhibitor and Cucurbitacin I inhibit viability of GBM cells **(A-B)** Cell Viability assay on U87 and T98G GBM cells using varying concentrations of BMS-5 measured over 5 days. **(C-D)** Cell Viability assay on U87 and T98G GBM cells using varying concentrations of Cucurbitacin I measured over 5 days. **(E-F)** Activated Cleaved Caspase 3/7 assay measuring cell apoptosis in presence of inhibitors. Measurements were take after 72h of treatment with 10 uM BMS-5 or 100nM of Cucurbitacin *p<0.05, **p<0.001,***p<0.0001.

Having established that BMS-5 and Cucurbitacin I inhibit cofilin phosphorylation, we explored whether these compounds had anti-viability and pro-apoptotic effects. Using the MTT cell viability assay, we observed significant decrease in cell viability in U87 and T98G cells using doses of 10–20 μM BMS-5 from days 3–5 with no effect at day 1 (**Figure 5A-B, p<0.05**). Similarly, Cucurbitacin I treatment resulted in a significant reduction in cell viability in U87 cells and T98G cells from 100nM–10μM from days 3–5 (**Figure 5C-D, P<0.05**). Reduction of cell viability correlated with increased apoptosis as measured by cleaved caspase 3/7 enzyme-linked immunosorbent assay (ELISA) (**Figure 5E-F, p>0.05**). We previously demonstrated that normal human astrocytes (NHAs) do not express p-CFL and LIMK1 [13]. We tested several low and high doses of BMS-5 and Cucurbitacin I and observed no cytotoxic effect on NHAs at low doses suggesting our small molecule inhibitors act specifically on tumor cells expressing LIMK1 and pCFL. Interestingly, reduced cell viability was observed in NHAs at high doses outside the potential therapeutic range of these molecules (50 μM for BMS-5 and 20 μM for Cucurbitacin I) (**Supplemental Figure 1A-B**).

### BMS-5 and Cucurbitacin I inhibit GBM cell migration, invasion

**Figure 6:**
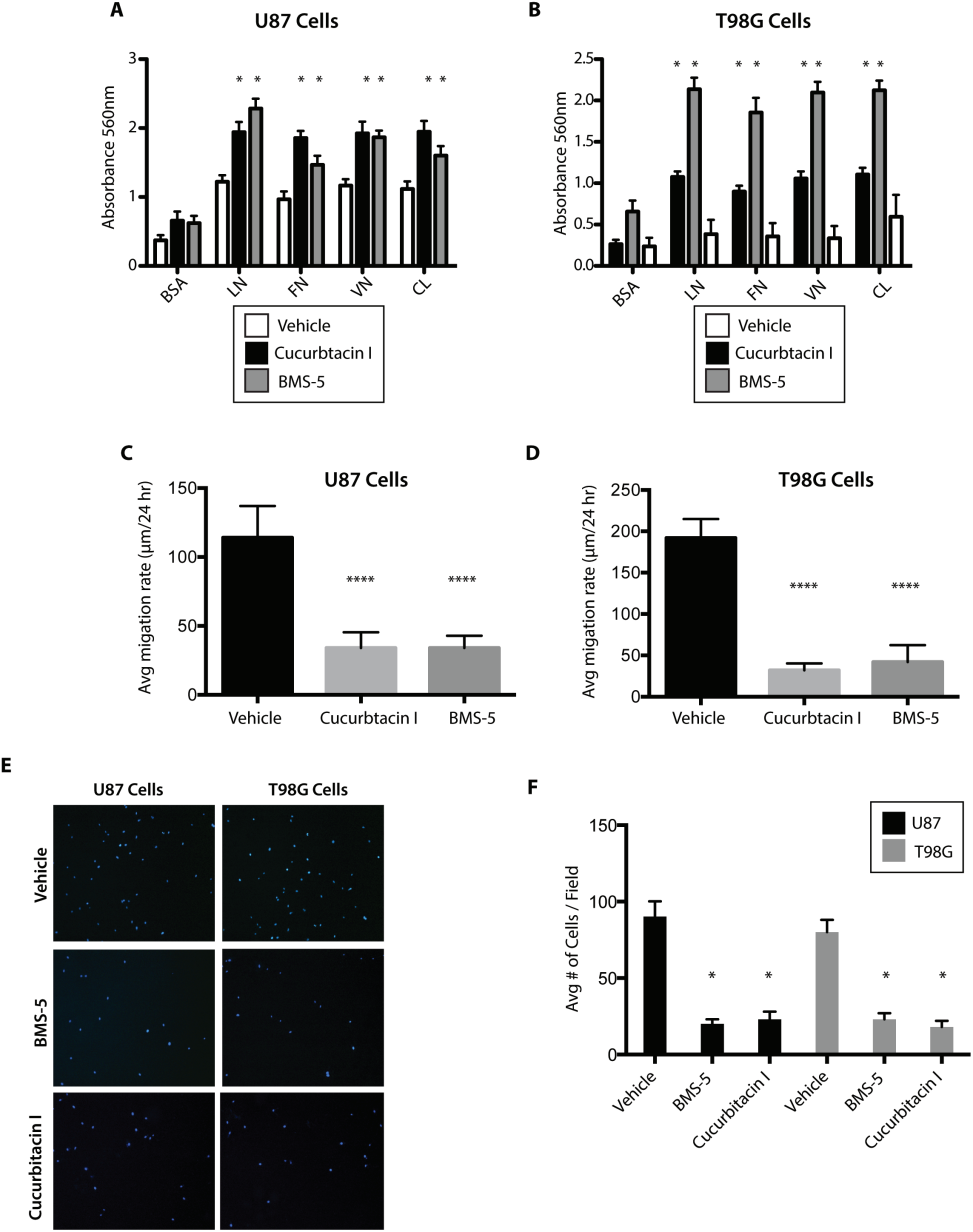
BMS-5 and Cucurbitacin I inhibit GBM cell migration, invasion **(A-B)** Adhesion assay of U87 and T98G cells after 12 of treatment with 10 uM BMS-5 or 100 nM cucurbitacin I on varying coated surfaces. BSA (Bovine Serum Albumin.Control), LN (Laminin), VN (Vitronectin), FN (Fibronectin), CL (Collagen). **(C-D)** Migration assay of U87 and T98G cells after 16H of treatment with 10 uM BMS-5 or 100 nM Cucurbitacin I. **(E)** Representative images of the invasion assay of U87 and T98G cells after 12H of treatment with 10 uM BMS-5 or 100 nM Cucurbitacin I. **(F)** Quantification of invasion assay of U87 and T98G cells after 12H of treatment with 10 uM BMS-5 or 100 nM Cucurbitacin I. *p<0.05,**p<0.001,***p<0.0001.

Having established that there was an anti-tumor effect on GBM cells with two small molecule inhibitors we queried whether there were pro-adhesion, anti-migratory and anti-invasion effects. Both BMS-5 (10 μM) and Cucurbitacin I (100 nM) promoted increased adhesion in U87 and T98G cells on various surface coatings (**Figure 6A-B,** p<0.05). Interestingly, BMS-5 and Cucurbitacin I reduced migration rates of both U87 and T98G cells (**Figures 6C-D**, p < 0.05). Next, to assess invasion, we performed a trans-well migration assay and observed significant reduction of invasion of T98G and U87 cells by BMS-5 and Cucurbitacin I (**Figure 6E-F**, p<0.05). The adhesion, migration and invasion assays were performed at time points less than 24 hr at which times the drugs do not alter cell number or viability.

## DISCUSSION

In this study, we have shown by transcriptional profiling of a dataset of over 400 genes that the CFL pathway is dysregulated in GBM, and that LIMK is upregulated in GBM using the REMBRANDT brain tumour dataset. From a prognostic perspective, patients with low LIMK1/2 expression had an overall better survival than those with high LIMK1/2 expression. Using a TMA with 20 patient matched samples of tumour core and rim regions, we demonstrate that LIMK1 and pCFL are more highly expressed at the tumour rim and periphery than in the core. We have shown that the LIM kinase inhibitor, Cucurbitacin, diminishes the phosphorylation of CFL at therapeutically achievable drug doses, and leads to decreased cellular proliferation, adhesion, and apoptosis. Finally, we have shown that BSM-5 and Cucurbitacin I are effective agents at inhibiting GBM cell migration and invasion. Taken together, these data support the notion that as direct upstream elements in the pCFL pathway, LIMK1/2 may be important regulators of GBM growth and invasion.

There are only two members of the LIM kinase family – LIM kinase 1 (LIMK1) and LIM kinase 2 (LIMK2). LIMK1/2 show unique structural arrangements with two LIM domains at the N-terminus, a PDZ domain connected to proline/serine-rich regions and a C-terminal kinase domain [14, 27]. The LIM domains enable the LIM kinases to interact directly with many macromolecular partners, including several of the Rho-GTPases such as Rac1, Cdc42, RhoA together with their downstream effectors PAK1-4, and ROCK1-2. The large number of molecular partners for LIMK helps to explain its role in a variety of cellular processes including cell migration, cancer cell invasion, metastasis, and neurodevelopmental disorders (e.g. William's syndrome) [28, 29].

LIMK1/2 are strategically located at a convergence point of upstream signals from a number of Rho GTPase family members [30]. The most important of these include Cdc42/Rac/Pak1, 2 and 4; Rho/ROCK/I&II; and Cdc42 MRCKa. From this convergence of signals, ROCKI and II, and PAK have been shown to phosphorylate and activate LIMK. LIMK1/2 then serve to phosphorylate CFL. However, there are phosphatases of CFL such as SSH and Chronophilin which counteract the phosphorylation and inactivation of CFL.

The gene for *LIMK1* is localized to 7q11.23, whereas the gene for *LIMK2* is found at 22q12.2 [31]. Mice in which *Limk1* is deleted show defects in synaptic structure and spine development [32]. Interestingly, Williams syndrome, a condition characterized by cognitive impairment, learning difficulties, and cardiovascular disease, is associated with gene deletions at 7q11.2 including the gene for *LIMK1* [29]. Mice with gene deletion of *Limk2* demonstrate impaired spermatogenesis [33]. When double *Limk1/Limk2* null mice are examined, these mice are viable, and show significant defects in synaptogenesis not seen in the single knockouts [32]. [34]

While the genes for *LIMK1/2* do not appear to be the target of mutations in human cancers, the subversion of their expression may be important in the process of cancer cell migration and metastasis [31]. In this regard, the balance between phosphorylated and unphosphorylated CFL, as would be regulated in part by LIMK1/2, may be critically important players. To support this concept, overexpression of LIMK1 has been described previously in malignant melanoma, breast cancer, and prostate epithelial cells/cancer [18, 21, 35]. When LIMK1 is overexpressed in breast cancer cells, increased invasion and metastasis of breast cancer cells are promoted [36]. Several reports have shown that modulating LIMK1 expression by antisense, by overexpression of LIMK1 by a dominant negative form, or by knockdown of LIMK2 by ribozyme-mediated knockdown will inhibit cancer motility, invasion, and metastasis [18, 36, 37].

There are a number of negative regulators of LIMK1/2. SSH reduces LIMK activity by dephosphorylation of transphosphorylated residues; LATS1 reduces LIMK1 activity; and Caspase 3 produces an inactive form of LIMK by truncation at Asp240 [38] [39]. To date, several small molecule inhibitors have been produced against the LIM kinases. These are generally phenyl-substituted primidines with 1-2 additional elements at the heterocyclic scaffold core [38, 39]. These were originally synthesized by Bristol Myers Squibb (BMS), and are known as the BMS compounds. Compound BMS-3 was shown to inhibit cofilin phosphorylation in a dose-dependent manner in MDA-MB-231 breast cancer cells [40]. It has also been used in MCF7 breast cancer cells and shows sensitization to doxorubicin, thus paving the way towards significant potential for more effective drug combination therapy [41]. In our study, we used BMS-5 which is a LIMK inhibitor without kinase selectivity [38]. The issue with some of the BMS compounds is their adverse effects on microtubule formation [41]. Cucurbitacin belongs to a class of biochemical compounds that plants such as pumpkins and gourds use to defend themselves against herbivores [42]. They are classified as steroids and often occur as glycosides. Structurally, Cucurbitacins are found as tetacyclic cucurbitane nuleus skeletons (triterpenes). Many of the cucurbitacins are known to be cytotoxic. Of the more than 30 cucurbitacins that have been identified, the ones most widely used for *in vitro* and *in vivo* cancer inhibition studies have been Cucurbitacin E, B, D, and I [42]. In our study we used Cucurbitacin I and noted a significant anti-tumour effect *in vitro* [42]. Unlike BMS-5, Cucurbitacin is not as selective an inhibitor of LIMK given its known effects on the signal transducer and activator of transcription (STAT), cyclo-oxygenase-2, and the apoptosis machinery. Recently, Cucurbitacin I was shown to inhibit JAK2/STAT3 and induce autophagy and apoptosis in glioblastoma cells *in vitro* and in flank xenograft studies [43]. These results indicate that Cucurbitacin I likely inhibits multiple signaling pathways and have functional effects on glioma cells in addition to its inhibition of migration and invasion described in the current study.

One of the major hurdles to treating GBM is failure of promising drugs to cross the BBB. Accordingly, we utilized a Multi-Parameter Optimization (MPO) algorithm to identify compounds with the physical properties to penetrate the BBB [44]. With the data from the MPO algorithm, BMS-5 and Cucurbitacin are predicted to have suboptimal BBB penetration in their native state (data not shown). Although beyond the scope of the current study manuscript, BMS-5 and Cucurbitacin could be delivered at higher concentrations to intracranial gliomas using techniques which focally disrupt the BBB such as Magnetic Resonance guided Focused ultrasound (MRgFUS) as we have shown in previous studies [45–47].

While the use of targeted Rho-GTPase inhibitors has not become mainstream therapy for any cancer at this time, there are several potential opportunities to increase their usage against human cancers. These include targeting the Rho activation with GEF inhibitors, inhibiting Rho-associated kinase activity, inhibiting MRCK activity, and p21-activated kinase activity. In combination, they may hold more promise than as stand-alone agents given the known redundancy of the elements in this system.

## MATERIALS AND METHODS

### Cell lines and cell culture and inhibitors

Human glioma cell lines T98G and U87 were purchased from the ATCC (American Type Culture Collection) and were maintained in Dulbecco's modified Eagle's medium (DMEM) supplemented with 10% of heat-inactivated fetal bovine serum (FBS) and cultured at 37°C, 5% carbon dioxide in a humidified chamber. NHAs were purchased from Lonza (Lonza, Walkersville, MD) and maintained in Astrocyte Growth Media (Lonza) con-taining 2.5% FBS, 0.1% ascorbic acid, 0.5% recombinant human epidermal growth factor, 0.1% GA-1000, 0.25% insulin, and 1% L-glutamine (Lonza). LIMK inhibitor was purchased from SYNKINASE (SYN-1024 BMS-5) and Cucurbitacin I was purchased from Sigma-Aldrich (C-4493). Both drugs were dissolved in DMSO making a master stock of 1 mM and used at doses and times points listed in the Results section.

### Western blot analysis

Cell lysates were prepared by harvesting cells in RIPA lysis buffer (Sigma-Aldrich, St. Louis, MO) with a cocktail of protease inhibitors (Roche Diagnostics, Indianapolis, IN). Protein concentration was determined using the Pierce BCA Protein Assay kit (Thermo Scientific, Rockford, IL) as per the manufacturer's protocol, and 30 μg of protein extracts were mixed with 6X SDS sample buffer (Tris pH 6.8, 1.7% SDS, glycerol and β-mercaptoethanol). Lysates were resolved on a 10% SDS-polyacrylamide gel of 1.5 mm thickness. Proteins were then transferred onto polyvinylidene fluoride transfer membranes (Pall Corporation, Pensacola, FL), and subsequently blocked with 5% skim milk in TBS-T adjusted to pH 7.4 (20 mM Tris aminomethane, 150 mM NaCl and 0.05% Tween 20) for 1 hour at room temperature. The membranes were immunoblotted overnight at 4°C with primary antibodies. After incubation with a primary antibody, the membranes were incubated at room temperature for 1 hour with a secondary antibody, horseradish peroxidise-conjugated Protein A (1: 5000; GE Healthcare, Buckinghamshire, UK) or a horseradish peroxidise-conjugated goat anti-rabbit immunoglobulin G antibody (1:5000; Cell Signaling Technology), and bound primary antibodies were visualized using Western Lightning Plus-ECL (PerkinElmer Inc., Waltham, MA). Relative densitometric ratios of average expression of LIMK1/2, CFL, and pCFL to that of actin are shown and were quantified using chemiluminescence signal in the linear range on an Alpha Imager imaging system. Antibodies used were as follows: Phospho Cofilin (p-serine 3): Rabbit polyclonal: 1:5000: SAB4504370-100UG. Cofilin: Rabbit polyclonal: 1:5000 Sigma-Aldrich SAB4500148-100UG. LIMK1: Rabbit polyclonal: 1:1000 Sigma-Aldrich HPA028516-100UL. LIMK2: Rabbit polyclonal: 1:1000 Sigma-Aldrich SAB4501760-100UG β-actin:Rabbit polyclonal 1:20000.

### Caspase 3/7 Assay

Caspase 3/7 activity levels as a measure of apoptosis were measured using the Apo-One^®^ Homogeneous Caspase 3/7 assay (Promega Corp., USA) that provides a profluorescent substrate and a cell lysis/activity buffer for Caspase 3/7 (DEVDase) activity. Briefly, 5,000 cells were seeded into 96 well plates and treated with varying doses of LIMK inhibitor BMS and Cucurbitactin I. After 48–72 hours of incubation with drug, 100 μL of Apo-One was seeded in each well, incubated for 3 hours and then fluorescence levels measured (485Ex/527Em).

### Cell proliferation assay

Cell proliferation was assessed by the colorimetric MTS assay using the Promega CellTiter 96 AQueous One Solution Cell Proliferation Assay (Promega, Madison, WI). Cells were seeded on 96-well plates at a density of 1000–5000 cells in 100 μl of culture medium containing 10% FBS per well. After one hour of incubation with 20 μl of MTS reagent per well, absorbance at 490 nm was measured using the VERSA max microplate reader (Molecular Devices, Sunnyvale, CA) at the specified time points. Experiments were repeated three times with eight replicates.

### Cell adhesion assay

96-well plates were coated with different ECM proteins (laminin [LN] and fibronectin [FN] at 10 μg/ml, collagen type IV [CL] at 50 μg/ml, and vitronectin [VN] at 1μg/ml) and 0.1% bovine serum albumin (BSA) in PBS at 4°C overnight. Purified human ECM proteins – LN, CL, VN and FN – were purchased from Sigma-Aldrich. The unbound sites were blocked with 0.1% BSA in PBS for one hour at 37°C. Cells treated with Cucurbitacin I and LIMK inhibitor were collected 12 hours after treatment and were resuspended in serum-free medium and plated on the coated plate at 5×10^4^ cells per well. After 3 hours incubation at 37°C in a 5% carbon dioxide humidified chamber to allow cells to adhere, unattached cells were removed by washing with PBS, and the remaining cells attached to the ECM on the bottom of the plates were fixed with 4% PFA for 15 minutes at room temperature. After washing with PBS three times, cells were stained with 0.5% crystal violet for 10 minutes. The excess stain was washed with water and cells were allowed to air-dry overnight. The crystal violet bound to the attached cells was solubilized in 200 μl of 1% SDS per well, and absorbance at 595 nm was measured using the VERSA max microplate reader (Molecular Devices). Experiments were repeated three times with five replicates.

### Radial cell migration assay

Cell migration was assessed by the microliter-scale radial monolayer assay using 10-well Teflon-coated glass slides (CSM Inc., Phoenix, AZ). Cells treated with Cucurbitacin I and LIMK inhibitor were collected 12 hours after siRNA transfection and were seeded through a cell sedimentation manifold (CSM Inc.) at 3000 cells per well. After 6 hours of incubation, manifolds were removed, and a best-fit circle circumscribing the cells was drawn as baseline. The cells were allowed to migrate for 24 hours in culture medium, and another circle circumscribing the newly migrated cells was drawn. The average migration rate (μm/24 hours) was determined by the increase of the diameter of the circle beyond the baseline diameter of the cells during a 24-hour period. Experiments were repeated three times with six replicates.

### Cell invasion assay

Cell invasion ability was assessed using BD BioCoat Matrigel Invasion Chambers consisting of Transwell-precoated Matrigel membrane filter inserts with 8 μm pores in 24-well tissue culture plates (BD Biosciences, Bedford, MA). 5×10^4^ of cells treated with both inhibitors were collected 12 hours after treatment and seeded onto the top of the chamber in DMEM with 0.1% FBS, and the bottom chamber was filled with DMEM with 10% FBS as a chemoattractant. The cells were incubated for 12 hours at 37°C in a 5% carbon dioxide humidified chamber to allow them to migrate through the filter membrane. After 12 hours of incubation, non-invading cells were removed from the upper surface of the membrane using cotton swabs, and the filter membranes were fixed with 4% PFA for 15 minutes. Subsequently, the filter membranes were mounted on glass slides using Vectashield with DAPI (Vector Laboratories Inc., Burlingame, CA) for nuclear staining. Fluorescence was visualized and images were taken using a fluorescence microscope and quantified using Image J software. Experiments were repeated three times in triplicate.

### Tissue microarray and immunohistochemistry

A glioma tissue microarray (TMA) containing tumour core (TC) and periphery (P) samples go GBM was obtained from Dr. Michael Berens (TGen, Phoenix AZ). The TMA used in this study consisted of a total of 20 matched TC and P GBMs spotted as 1 mm cores.

The TMA and GBM sample slides were deparaffinized in xylene and rehydrated in ethanol. Then, antigen retrieval was achieved by microwave pressure-cooking in sodium citrate solution adjusted to pH 6.0, followed by blocking of endogenous peroxidase activity using 3% hydrogen peroxide in methanol for 20 minutes. Blocking was performed using 10% donkey serum for one hour at room temperature. The tissues were incubated with a rabbit polyclonal p-CFL antibody (Sigma-Aldrich, SAB4504370-100UG) and anti-LIMK1 antibody (Sigma-Aldrich HPA028516-100UL) both at 1:100 dilutions at 4°C overnight. Subsequently, the tissues were incubated with a biotinylated secondary antibody (Vector Laboratories Inc.) for one hour at room temperature. For detection, the ABC kit (Vector Laboratories Inc.) and DAB chromagen (Sigma-Aldrich) were used. Finally, the tissues were counterstained in hematoxylin (Fisher Scientific Inc., Fair Lawn, NJ), dehydrated in ethanol, washed in xylene, and mounted on glass slides using Permount (Fisher Scientific Inc.). P-CFL and LIMK1 positivity and staining were independently and scored by neuropathologist Dr Sidney Croul, who was blinded to the origin of the samples.

### Microarray and Copy Number Data

Glioma and GBM mRNA and copy number expression data was obtained from the Repository of Molecular Brain Neoplasia Data (REMBRANDT, https://caintegrator.nci.nih.gov/rembrandt/) [25] and analysis was performed using Rembrandt online statistical software. Normal brain versus mesenchymal GBMs were obtained from the TCGA data portal (https://tcga-data.nci.nih.gov/tcga/) including 10 normal brain and 51 mesenchymal GBMs as defined from the Verhaak et al. study [22]. Affymetrix gene expression profiles on the U133A platform were downloaded as raw CEL files. CEL files were then imported into Affymetrix Expression Console (Version 1.1) and gene level analysis (CORE content) was performed. Arrays were quantile normalized (RMA sketch normalization) and summarized using PLIER with PM-GCBG background correction followed by a COMBAT analysis (combatting batch correction) using the statistical software from the Broad Institute (Gene-Pattern). Probesets were annotated according to the human genome build HG19 (GRCh37). Gene lists involving cell migration and invasion were extracted for differential gene expression analysis using gene-ontology terms (GO-terms) for invasion and migration: Invasion (GO:0044409) Neuron Migration (GO:0001764), Glial Migration (GO:0008347), Mesenchyme Migration (GO:0090131), forebrain cell migration (GO:0021885), cerebral cortex migration (GO:0021795). Using these terms we generated a list of 400 genes involved in core invasion and migration processes. Significant genes were identified as genes differentially expressed by 1.5 fold, having a q-value less than 5% (false discovery rate) and a p-value less than 0.05 using a significance of microarray analysis (SAM) pairwise test.

### Bioinformatic and Statistical analysis

Experiments were performed in triplicate unless otherwise denoted with mean and standard error of the mean reported where appropriate. Where appropriate, direct comparisons were conducted using an unpaired two-tailed Student's t-test. Analysis of variance (ANOVA) was conducted for multi-group comparisons followed by a post-Dunnetts test (groups compared to one control group) or post-Tukey (to identify differences among sub-groups). Significance was defined as p < 0.05. Kaplan-Meier survival curve analysis was performed and the survival was statistically compared by the log-rank test. A probability value less than 0.05 was considered significant.

Blood brain barrier permeability predictions were performed using the CNS multiparameter optimization (CNS MPO) algorithm, an *in silico* BBB permeability prediction model used to prioritize compounds for drug development in CNS diseases [44]. Physiochemical properties used in the analysis were estimated using Advanced Chemistry Development Labs (ACD/Labs) PhysChem Suite.

## SUPPLEMENTARY FIGURE


