# Supplementary material for: Transcriptional profiling of GBM invasion genes identifies effective inhibitors of the LIM kinase-Cofilin pathway
Source: Oncotarget. 2014 Sep 5;5(19):9382–95. doi: 10.18632/oncotarget.2412 (PMC4253441; doi:10.18632/oncotarget.2412)
Supplement: Supplementary file 1 [file oncotarget-05-9382-s001.pdf]

SUPPLEMENTARY FIGURE

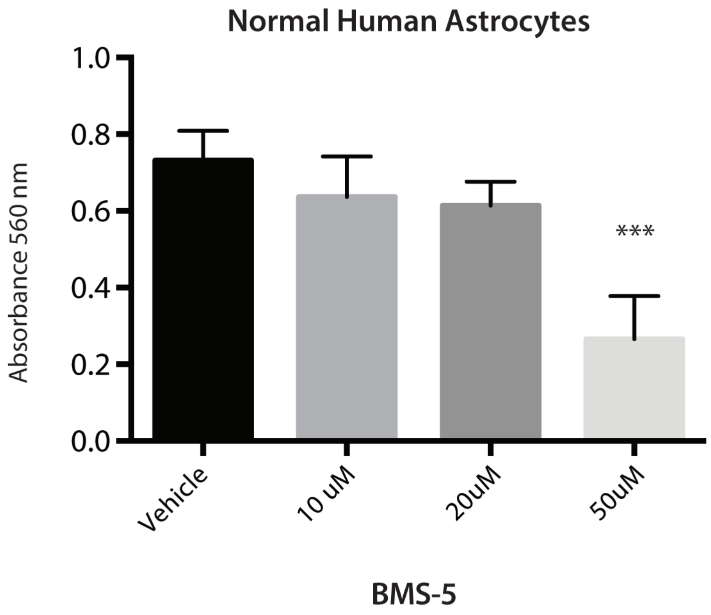

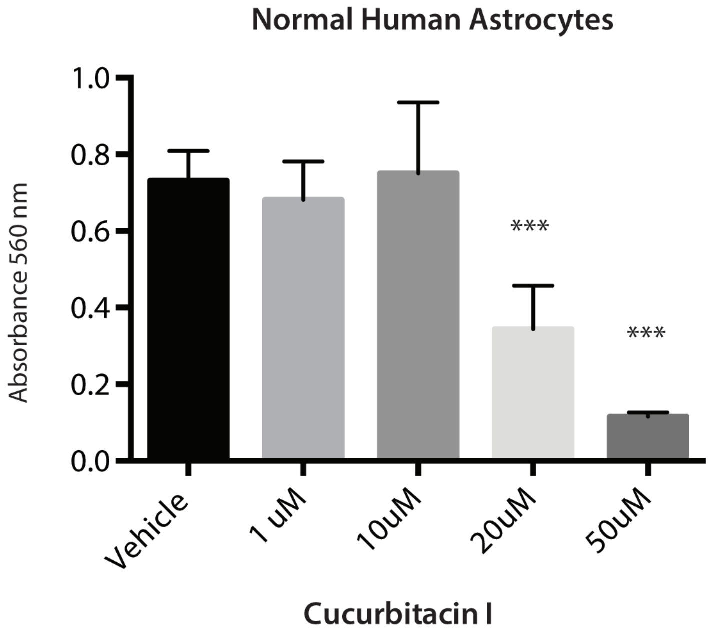

Supplementary Figure S1: (A) Cell Viability assay on normal human astrocytes using varying concentrations of BMS-5 measured on day 5. **(B)** Cell Viability assay on normal human astrocytes using varying concentrations of Cucurbitacin I measured on day 5. ***$p<0.0001$.
